# Allergic diseases of the skin and drug allergies – 2013. Longitudinal analysis of fecal microbiota of infants followed-up for eczema till 2 years of age

**DOI:** 10.1186/1939-4551-6-S1-P100

**Published:** 2013-04-23

**Authors:** Gaik Chin Yap, Christophe Lay, Marion Aw, Lynette Shek Pei-Chi, Yudong Zhao, Doreen Leow, Bee Wah Lee

**Affiliations:** 1 National University of Singapore, Singapore; 2 Danone Research-Centre for Specialised Nutrition, Singapore; 3 Paediatrics, National University of Singapore, Singapore; 4 Singapore Clinical Research Institute, Singapore

## Background

Studies have suggested that selective microbial targets prevail in the fecal microbiota of infants with eczema. This study aims to evaluate and compare the composition of fecal microbiota of infants who developed eczema by 2 years of age and healthy controls.

## Methods

Children with eczema at 2 years old (n=26) and their matched (for gender, mode of delivery and feeding in first 6 months) healthy controls (n=26) were selected from the placebo group of a cohort of at-risk infants participating in an randomized double-blind placebo controlled trial on the protective effects of supplemental probiotics (first 6 months) on eczema and allergies. Children with eczema were subclassified into atopic eczema (n=12) and non-atopic eczema (n=14). Molecular evaluation of fecal microbiota were conducted using Fluorescence In Situ Hybridization-Flow Cytometry (FISH-FC) for fecal samples collected at 3 days, 1, 3, and 12 months. Probes were selected to target *Eubacterium rectale-Clostridium coccoides* group (Erec482), *Clostridium leptum* subgroup (Clep866 and the corresponding competitor probes), *Bacteroides-Prevotella* group (Bac303), *Bifidobacterium* genus (Bif164), *Atopobium* group (Ato291), *Lactobacilli- Enterococci* group (Lab158), Enterobacteriaceae family (Enter1432) and *Clostridium perfringens* (Cperf191). Linear mixed model was used to evaluate the longitudinal differences (i.e. 4 time points) of bacterial targets while adjusting for gender, mode of delivery, feeding up to 6 months, and allergic rhinitis and wheezing within the eczema group at 2 years of age.

## Results

Longitudinal analyses over four time points showed that higher relative abundance of Enterobacteriaceae [coefficient (B): 1.104, 95% confidence interval (CI):0.175-2.033, adj p=0.022] in children with eczema by 2 years of age. Similar observations were made when eczema group was subanalyzed into non-atopic and atopic eczema, where higher relative abundance of Enterobacteriaceae [B:1.357, 95%CI; 0.382-2.332, adj p=0.008] and [B:1.165, 95%CI; 0.221-2.109, adj p=0.019] were observed respectively as compared to healthy controls. Relative abundance of *Clostridium perfringens* were also higher when subanalyzed for non-atopic [B:0.572, 95%CI; 0.0009-1.144, adj p=0.050] and atopic eczema [B:0.000451, 95%CI: 0.0001-0.0007, adj p=0.012] compared to healthy controls.

## Conclusions

Our data suggests that relative abundance of selective microbial targets particularly Enterbacteriaceaea and *Clostridium perfringens* in the fecal microbiota of infants influence the development of eczema in early childhood.

